# Inflammatory markers in a 2-year soy intervention among premenopausal women

**DOI:** 10.1186/1476-9255-6-9

**Published:** 2009-04-07

**Authors:** Gertraud Maskarinec, Jana S Steude, Adrian A Franke, Robert V Cooney

**Affiliations:** 1 Cancer Research Center of Hawaii, Suite 510, 1236 Lauhala Street, Honolulu, Hawai'i 96813, USA

## Abstract

**Background:**

Epidemiologic evidence supports a role of soy foods in breast cancer etiology. Because chronic inflammation appears to be a critical component in carcinogenesis, we examined the potential anti-inflammatory effects of soy foods.

**Methods:**

The original 2-year dietary intervention randomized 220 premenopausal women of whom 183 women (90 in the intervention group and 93 in the control group) were included in the current investigation; 40% were of Asian ancestry. The intervention group consumed two daily soy servings containing 50 mg of isoflavones (aglycone equivalents), whereas the controls maintained their regular diet. Five serum samples obtained at month 0, 3, 6, 12, and 24 were analyzed for interleukin (IL)-6, C-reactive protein (CRP), leptin, and adiponectin by ELISA. For statistical analysis, mixed models were applied to incorporate the repeated measurements.

Results:

The levels of all analytes were lower in Asian than Caucasian women. Overweight women had significantly higher levels of CRP, IL-6, and leptin and lower levels of adiponectin than normal weight women. We did not observe a significant effect of soy foods on the four markers, but leptin increased in the control and not in the intervention group (*p *= 0.20 for group-time effect); this difference was significant for Asian (*p *= 0.01) and obese women (*p *= 0.005).

**Conclusion:**

During this 2-year intervention, soy foods did not modify serum levels of CRP, IL-6, leptin, and adiponectin in premenopausal women although leptin levels remained stable among women in the intervention group who were obese or of Asian ancestry. Further studies with diverse markers of inflammation are necessary to clarify the specific effect of soy on immune responses.

## Introduction

Isoflavones, weak estrogenic compounds found in high concentrations in soy beans, have been explored as cancer preventive agents for a long time [1,2]. A meta-analysis that described a 15% lower breast cancer risk related to soy [3] offers fairly strong epidemiologic support for a protective effect of soy that, however, might be restricted to Asian populations [4]. Different hypotheses about the underlying mechanism of cancer-protective effects of soy have been evaluated, including antioxidant, antiproliferative properties, and the modulation of lipoprotein metabolism, as well as its estrogenic and antiestrogenic effects [5]. The results of a 2-year soy intervention at our center [6] and reports from other trials [7-10] indicate that the preventive effects of soy on breast cancer risk, if they exist, are not mediated by their effects on the major circulating sex hormones. On the other hand, results from animal studies and human trials support the hypothesis that soy or isoflavones reduce chronic inflammation, a possible risk factor for breast cancer [11-13]. Circulating inflammatory markers, such as cell-adhesion molecules and C-reactive protein (CRP), were decreased in some studies [14,15]. Genistein appears to down regulate the inflammatory response through its tyrosine kinase inhibitory effects [16,17].

While the original objective of our randomized trial had been to examine effects of two daily soy servings on steroid hormones and mammographic densities [6,18], the aim of the current study was to examine the effects of soy foods on serum markers of obesity and systemic inflammation, interleukin (IL)-6, IL-2, CRP, leptin, and adiponectin, in premenopausal healthy women. The choice of markers was driven by the concept of capturing different aspects of the immune response through a combination of cytokine levels as it appears that they predict disease better than single markers [19]. IL-6 is one of the major components responsible for the acute phase protein synthesis by the liver, particularly CRP, a sensitive, non-specific indicator of inflammation. IL-2 is a growth factor for antigen-stimulated T lymphocytes, where it increases cytokine synthesis and B cell proliferation [20]. Leptin, a possible link between nutritional status and the immune function, is a marker of obesity, participates in pro-inflammatory responses, and is an important growth factor for breast cancer [20,21]. Adiponectin is the most abundant protein in adipocytes with a strong anti-inflammatory function in addition to its anti-atherogenic and insulin-sensitizing properties [22].

## Methods

### Study design

As described elsewhere [6], the participants were recruited by sending out 10,022 invitations to women who had received a normal screening mammogram. Of these, 975 (9.73%) interested women replied and 352 eligible women aged 35–46 years were identified during a telephone-screening interview. Women were excluded from this study due to use of oral contraceptives or other sex hormones, diagnosis of cancer, hysterectomy, and no intact ovary or no regular menstrual periods. After a run-in period, 220 women were randomized to a soy diet or to the control group and 189 subjects completed 2 years of intervention [6]. The Institutional Review Boards of the University of Hawaii approved the study protocol; participants signed informed consent and gave written permission to use frozen samples for future analyses.

### Study procedures

This trial was designed to provide two servings of soy per day containing approximately 25 mg aglycone equivalents of isoflavones per serving [23]. A choice of tofu, soy milk, roasted soy nuts, soy bars, and soy protein powder was offered to the participants. The same brands of soy foods was used throughout the intervention and the isoflavone content of food items was monitored by high-pressure liquid chromatography (HPLC) with photo diode array detection [24]. Women in the control group were instructed to maintain their regular diet. Body weight was recorded at baseline and at each study visit. Adherence to the study protocol was high in both arms of this study [6,25]. Subjects in the intervention group showed an increase in self-reported intake of isoflavones from 4.4 mg/day at baseline to 57.3 mg/day as assessed by unannounced 24-hour recalls, which was confirmed by an increase in urinary isoflavone excretion from 8.0 to 59.8 nmol/mg creatinine. Controls continued their usual soy intake as confirmed by 24-hour recalls and urinary isoflavone excretion.

### Serum Sample Collection

This study makes use of existing serum samples that were collected during the 2-year trial period. We collected blood samples at baseline as well as 3, 6, 12 and 24 months after randomization timed to occur 4–6 days after ovulation as determined by an ovulation kit [6]. Blood was allowed to clot for 30 minutes and centrifuged at 3000 rpm for 15 minutes; aliquoted serum was frozen at -80°C. Not all women donated five samples because of dropout or failure to provide a specimen at one point of the study. We analyzed samples from 183 women, 93 in the control group and 90 in the intervention group. For 141 women, 5 specimens were available. There were 4 samples available for 37 women and only 3 measurements for 5 women, but each of these women provided a baseline sample.

### Analytical methods

Serum levels of IL-6, IL-2, leptin, and adiponectin were assessed by double-antibody enzyme-linked-immunosorbend-assay ELISA assays (R&D Systems, Minneapolis, MN) according to the manufacturer's specifications. Plots of concentration vs. absorbance for standards were prepared using a four parameter fit and concentrations of unknown samples extrapolated from the standard curve. The CRP assay was based on a latex particle enhanced immunoturbidimetric method using a Cobas MiraPlus clinical autoanalyser and a kit from Pointe Scientific, Inc, Lincoln Park, MI. Batches of 30 or 40 samples contained all 5 samples of each woman and an equal number of women by group. Detection limits were 0.1 mg/L (CRP), 0.2 pg/mL (IL-6), 0.8 ng/mL (leptin), 1.8 μg/mL (adiponectin) and 31 pg/mL (IL-2). Positive IL-2 values could only be detected in 6 women and were, therefore, not included in the analysis. The assay quality was assessed by 49 blinded controls from a pooled sample donated by 10 premenopausal center employees. The mean intra-batch coefficients of variation (CV) for CRP, leptin, adiponectin, and IL-6 were 6.1%, 4.6%, 14.0% and 6.8%, whereas inter-batch CVs were 18.2%, 9.5%, 24.9% and 17.8%, respectively. Urinary isoflavone excretion was measured by HPLC and serum estradiol by radioimmunoassay as described previously [6].

### Statistical Analysis

Statistical analyses were performed using the SAS statistical software package version 9.1 (SAS Institute, Inc., Cary, NC). Non-normally distributed data were log-transformed prior to statistical analysis (see table footnotes for details). To assess differences in baseline characteristics between the two groups, Student's *t *tests were performed for continuous variables and χ^2 ^tests for categorical variables. We calculated unadjusted means and standard deviations for each marker by group and time of blood draw. To assess the correlation of markers, we computed Spearmen correlation coefficients (r_s_); to measure stability over time, we calculated intraclass correlation coefficients (ICC).

To assess the effect of the soy diet on the markers, we examined overall group mean differences using the Proc Mixed procedure in SAS 9.1; the repeated measurements were included as random effect [26,27]. Mixed models address the dependence of observations in a repeated measurement design by modeling the within-person and between-person variances simultaneously. They allow for an analysis of repeated measures with unbalanced times of measurement; subjects contribute to the overall model as long as they have one measurement [28]. We examined the significance of the dietary intervention, the change over time, and the interaction between group assignment and time. To assess their possible influence on the intervention, body mass index (BMI), serum estradiol, and urinary isoflavone excretion were included as time dependent covariates. Analyses were repeated for subgroups after stratification by ethnicity (Asian vs. Caucasian) and BMI (<25, 25–30, and >30 kg/m^2^).

## Results

This study included 67 Caucasians, 49 Japanese, 23 Hawaiians, 11 Filipinos, 14 Chinese, and 19 women of mixed and other ethnicities. Women in the intervention group (n = 90) did not differ significantly from women in the control group (n = 93) (Table 1). The mean age at baseline was 42.7 years in the intervention and 43.4 years in the control group (*p *= 0.21). Body weight at baseline increased in both groups throughout the trial: 0.8 kg in the diet and 1.2 kg in the control group (*p *= 0.53). The weight gain was slightly higher among Asians than non-Asians (1.4 vs. 0.7 kg; *p *= 0.27). The mean increase among Asian controls vs. intervention women was 1.1 vs. 1.7 kg (*p *= 0.46); the respective values for non-Asians were 0.6 vs. 0.8 kg (*p *= 0.80).

**Table 1:** Characteristics of study participants in a 2-year soy trial

**Variable**	**Controls**	**Intervention**	***P*-value^2^**
		
	**Mean [SD]**	**Mean [SD]**	
Number obf subjects	93	90	
Age at randomization (y)	42.7 [3.0]	43.3 [2.9]	0.21
Ethnicity^1^			
Caucasian	35 [37.6%]	32 [35.6%]	0.96
Japanese	24 [25.8%]	25 [27.8%]	
Hawaiian	11 [11.8%]	12 [13.3%]	
Filipino	7 [7.5%]	4 [4.4%]	
Chinese	7 [7.5%]	7 [7.8%]	
Mixed/other	9 [9.6%]	10 [11.3%]	
Body weight (kg)			
At baseline	68.3 [17.5]	67.9 [15.2]	0.87
After 2 y	69.1 [16.1]	69.1 [15.5]	0.99
Change over 2 y	0.8 [4.1]	1.2 [3.6]	0.54
Body mass index (kg/m^2^)			
At baseline	26.1 [6.1]	26.2 [5.4]	0.96
After 2 y	26.4 [6.5]	26.6 [5.5]	0.85
Change over 2 y	0.32 [1.5]	0.45 [1.4]	0.54
Lifetime soy intake (svgs)	2440 [2967]	2044 [2541]	0.33
Soy consumption (svgs/y)^1^			
During early life >0	49 [52.7%]	47 [52.2%]	0.95
During adult life >36	47 [50.5%]	45 [50.0%]	0.94
Lifetime ≥ 27	49 [52.7%]	47 [52.2%]	0.95
Marker levels at baseline			
CRP (mg/L)	2.31 [3.28]	1.95 [3.10]	0.44
IL-6 (pg/mL)	1.36 [1.47]	1.06 [0.64]	0.08
Adiponectin (μg/mL)	8.13 [3.68]	7.87 [4.13]	0.65
Leptin (ng/mL)	19.06 [15.78]	19.45 [15.02]	0.87

^1 ^Frequencies and percentages

^2 ^p-values from χ^2^- and Student's *t *test

The ICCs for leptin, CRP, IL-6, and adiponectin based on all measurements over 2 years were 0.91, 0.65, 0.71, and 0.84, respectively. Although IL-6 and CRP levels were slightly higher in the control than in the intervention group, the mean levels at baseline did not vary significantly by group; the respective *p*-values were 0.08 and 0.44. CRP and IL-6 were strongly correlated (r_s _= 0.54; *p *< 0.0001). Leptin was also associated with IL-6 (r_s _= 0.50; *p *< 0.0001) and CRP (r_s _= 0.49; *p *< 0.0001). We found inverse correlations of adiponectin with IL-6 (r_s _= -0.29; *p *< 0.0001), CRP (r_s _= -0.15; *p *< 0.0001) as well as leptin (r_s _= -0.26; *p *< 0.0001). Mean levels of all analytes were lower in Asian than Caucasian women at baseline: 1.48 vs. 2.57 mg/l for CRP (*p *= 0.01); 1.04 vs. 1.32 pg/mL for IL-6 (*p *= 0.09); 7.52 vs. 8.33 μg/mL for adiponectin (*p *= 0.17), and 14.8 vs. 22.3 ng/mL for leptin (*p *= 0.0004). Furthermore, overweight women (BMI ≥ 25 kg/m^2^) had significantly higher levels of CRP (3.4 vs. 1.0; *p *< 0.0001), IL-6 (1.5 vs. 0.9; *p *< 0.0001), and leptin (28.3 vs. 10.7; *p *= 0.001) than normal weight women, whereas adiponectin was higher in normal than overweight women (9.2 vs. 6.7; *p *< 0.0001).

We did not observe a significant effect of the soy intervention on any of the analytes (Table 2). The respective *p*-values for the interaction between group assignment and time were 0.48 for CRP, 0.51 for IL-6, and 0.71 for adiponectin. However, a non-significant effect of the soy diet was observed on leptin levels. While leptin levels were stable in the intervention group, they increased over time from 19.1 ng/mL to 21.0 ng/mL in the control group (*p *= 0.03 for time, *p *= 0.20 for group-time interaction and *p *= 0.43 for group effect).

**Table 2:** Effects of a 2-year soy trial on serum levels of inflammatory markers^1^

**Marker**	**Time (months)**	**Control**			**Intervention**			***P*-values**		
		**N**	**Mean**	**SD**	**N**	**Mean**	**SD**	**Group**	**Time**	**Interaction**
CRP (mg/L)	0	92	2.3	3.3	90	1.9	3.1			
	3	84	2.3	3.4	82	1.7	2.8			
	6	86	2.6	3.7	85	1.8	3.5			
	12	89	2.2	3.1	86	1.6	2.6			
	24	85	2.1	3.0	83	2.6	4.5	0.47	0.35	0.48

IL-6 (pg/mL)	0	93	1.4	1.5	90	1.1	0.7			
	3	84	1.5	1.8	82	1.1	0.5			
	6	88	1.3	1.0	86	1.2	1.0			
	12	89	1.3	1.2	86	1.1	0.6			
	24	85	1.3	1.2	83	1.2	0.9	0.21	0.15	0.51

Adiponectin (μg/mL)	0	93	8.1	3.7	90	7.9	4.1			
	3	84	8.2	3.5	82	7.8	4.3			
	6	89	8.4	4.0	86	8.2	4.5			
	12	89	8.3	4.0	85	7.6	4.0			
	24	85	8.4	3.9	83	7.9	3.9	0.61	0.88	0.71

Leptin (ng/mL)	0	93	19.1	15.8	90	19.4	15.0			
	3	84	20.0	17.3	82	19.6	15.2			
	6	88	20.7	16.8	86	19.6	14.9			
	12	89	21.1	17.1	86	19.4	14.3			
	24	85	21.0	18.0	83	19.2	14.9	0.43	0.03	0.20

^1^Obtained from mixed linear models; log-transformed values were used (except adiponectin) and back-transformed means are presented.

For IL-6, CRP, and adiponectin, stratification by ethnicity and BMI did not reveal a significant intervention effect for any subgroup (data not shown). For leptin, stratified post-hoc analysis of Asian women showed a significant intervention effect of soy (*p *= 0.01) that was not present among Non-Asians (Table 3). In the Asian controls, leptin levels rose from 12.6 to 14.7 ng/mL, whereas they remained stable in the intervention group (17.1 to 16.6 ng/mL). Including BMI as a time dependent variable in the models did not explain this change; the interaction effect remained significant (*p *= 0.006). Similarly, a significant effect of soy on leptin was observed in obese women (BMI ≥ 30 kg/m^2^) (*p *= 0.005) and could not be explained but BMI, but not in those with lower BMIs (Table 3). In the obese group, controls showed an increase in leptin from 38.7 to 47.6 ng/mL, whereas levels in the intervention group were stable (38.1 and 38.5 ng/mL).

**Table 3:** Effects of a 2-year soy intervention on leptin levels by ethnicity and body mass index^1^

**Subgroup**	**Time (months)**	**Control**			**Intervention**			***P*-values**		
		**N**	**Mean**	**SD**	**N**	**Mean**	**SD**	**Group**	**Time**	**Interaction**
Asian	0	38	12.6	8.5	36	17.1	12.5			
	3	33	13.5	10.6	31	16.5	14.0			
	6	36	15.0	11.0	33	15.0	8.0			
	12	38	15.0	10.5	34	16.3	12.4			
	24	35	14.7	9.9	33	16.6	14.3	0.06	0.10	0.01

Non-Asian	0	55	23.5	18.0	54	21.0	16.4			
	3	51	24.2	19.5	51	21.6	15.7			
	6	53	24.7	19.0	53	22.4	17.4			
	12	51	25.7	19.6	52	21.4	15.3			
	24	50	25.3	21.0	50	20.9	15.1	0.70	0.15	0.63

BMI <25 kg/m^2^	0	48	9.8	7.0	46	11.5	6.8			
	3	44	10.3	7.8	42	11.5	8.0			
	6	44	11.2	8.2	42	12.0	8.0			
	12	44	10.5	6.4	41	11.0	6.3			
	24	45	10.6	6.9	40	11.1	6.9	0.18	0.11	0.58

BMI 25–30 kg/m^2^	0	25	21.1	7.7	23	18.2	7.9			
	3	21	22.1	11.3	21	20.4	10.0			
	6	24	22.9	11.6	25	19.7	8.4			
	12	26	22.9	10.4	27	20.7	10.9			
	24	23	21.5	10.1	28	20.5	10.6	0.67	0.23	0.94

BMI >30 kg/m^2^	0	20	38.7	19.6	21	38.1	17.8			
	3	19	40.1	20.7	19	36.7	17.8			
	6	20	39.0	20.4	19	36.3	19.6			
	12	19	43.4	19.3	18	36.6	16.3			
	24	17	47.7	19.4	15	38.5	18.9	0.71	0.02	0.005

^1^Obtained from mixed linear models; log-transformed values were used and back-transformed means are presented.

Additional analyses by compliance and age did not suggest an effect in any subgroup. Urinary isoflavone excretion was not related to any of the four markers. However, serum estrogen levels showed a significant direct association with leptin only (*p *= 0.003). For none of the four markers were the intervention results modified by including estradiol or urinary isoflavone excretion into the mixed models. We also excluded women with CRP levels >3.0 mg/l, a level that most likely indicates an acute condition, but did not observe an effect of soy.

## Discussion

Contrary to our hypothesis, these results do not indicate a general intervention effect of a 2-year soy diet on CRP, IL-6, and adiponectin in premenopausal women. Although the overall effect was not significant for leptin, we noted that leptin levels remained stable in the intervention group over 2 years while they increased by 10% in the control group. This trend was statistically significant among women with Asian ancestry as well as in obese subjects. Over 2 years, leptin levels increased by 17% in the Asian controls and by 23% among obese controls, while levels in the intervention group decreased or remained stable. The differential effect was not explained by differences in weight gain and suggests that soy may prevent an increase in leptin over time due to weight gain. The stability of serum levels over time indicates the usefulness of these markers as a tool in epidemiological research.

Previous interventions show conflicting results and were limited by their short duration and their small sample size. Our results agree with several studies that report no effect of soy foods on CRP [13,29-34]. Yet, some reports described lower CRP levels with soy consumption [35-38]. IL-6 was measured in four investigations with little indication that supplementation had an effect [29,34,38,39]. Contrary to our findings, adiponectin levels increased in a soy intervention study among postmenopausal women [40], but no studies in premenopausal women have been performed to our knowledge. Two studies measuring leptin during an intervention with soy protein isolate reported no change in leptin levels over 3 months [40,41]. The investigation of vascular markers of inflammation also resulted in largely negative findings [13,15,32,38].

Given the multiple comparisons, the significant results for leptin among obese women could be a chance finding, but it is possible that soy only has a beneficial effect on these women due to their abnormally high leptin levels. This observation would correspond to the finding that soy foods have a more favorable effect among individuals with high than normal plasma lipid concentrations [42]. Whereas an increase in leptin among controls was expected as the result of the mean 1.2 kg weight gain, the stable levels in the intervention group, which also gained 0.8 kg over the 2 years, might suggest potential beneficial effects of soy on leptin. Furthermore, the effect may have been stronger in Asian women because of their higher percent body fat and their larger proportion of adipose fat tissue as compared to Caucasians [43]. These findings may contribute to the lower breast cancer risk associated with soy consumption [3], particularly for Asians [4], through a number of different mechanisms. As an adipocyte-derived cytokine, leptin acts pro-inflammatory through induction of cytokines by T cells [20], but it has also been identified as a growth factor for breast cancer [21]. It appears to increase estrogen levels by stimulating aromatase expression, and to activate estrogen receptors [21], and to induce growth in breast cancer cell lines and human primary breast carcinoma [44].

A number of reasons may explain the lack of an intervention effect. Due to limits of detection, availability of serum, and budgetary constraints, we were not able to measure more markers. The four measured markers, CRP, IL-6, leptin, and adiponectin, represent only a small subset of inflammatory processes. To assess the overall immune response and complex secretion and interaction patterns of cytokines, measuring many more cytokines would be useful [19]. Our choice of markers was based on their broad usage, detection levels, and availability of serum. Another limitation of our study design was that little attention was paid to acute infections and the use of NSAIDs or statins. As to adjustment for body fat, BMI is not a very representative measure across ethnic groups [43], but no other information on adiposity was available.

Our study had several strengths. We were able to use existing serum samples from a 2-year randomized intervention with excellent compliance [6] to investigate a relatively novel hypothesis. The serum samples were collected during the luteal phase under standardized conditions and analyzed with highly reproducible results. The long intervention period, the relatively large sample size, and the availability of multiple samples for each subject were a great benefit because the study design addressed the intraindividual variability in inflammatory markers. Also, traditional soy foods, as in our study, represent the nutritional exposure of habitual soy consumers in Japan in China with a low breast cancer risk more closely than protein isolates or isoflavone supplements used in most of the previous reports.

In contrast to the findings of this soy intervention that did not observe a change in serum levels of CRP, IL-6, leptin, and adiponectin during 2 years, experimental findings [11,12,17] and animal studies [45,46] support an influence of soy foods on immune responses. If an effect exists, it is possible that soy protein, n-3 fatty acids, a major component of soy beans that may influence immune responses [47], or other components rather than isoflavones are responsible for the biological effects of soy beans. Despite the overall negative findings of this report, it is possible that dietary patterns rich in soy food may have a beneficial effect on markers of obesity and/or chronic inflammation in specific populations. This hypothesis deserves further investigation, in particular the possible link between soy, leptin, and breast cancer.

## Abbreviations

BMI: Body mass index; CI: Confidence interval; CRP: C-reactive protein; CV: Correlation of variation; ICC: Intraclass correlation coefficient; IL: Interleukin

## Competing interests

The authors declare that they have no competing interests.

## Authors' contributions

GM conceived of the study, obtained funding, directed the statistical analysis, and finalized the manuscript. JSS carried out the statistical analysis and drafted the manuscript. AAF participated in the study design and conduct of the original trial; he also assisted in the selection of markers and the planning of the lab analyses. RVC was in charge of the ELISA assays and contributed to the data analysis. All authors read and approved the final manuscript.
